# Supplementary material for: Glycolysis-enhancing α1-adrenergic antagonists modify cognitive symptoms related to Parkinson’s disease
Source: NPJ Parkinsons Dis. 2023 Mar 2;9:32. doi: 10.1038/s41531-023-00477-1 (PMC9981768; doi:10.1038/s41531-023-00477-1)
Supplement: Supplementary file 1 — Supplemental Table 1 [file 41531_2023_477_MOESM1_ESM.pdf]

Supplemental Table 1: Cohort Summary Measures Before and After Matching. Values reported are median and inner quartile range for continuous variables or count and percent for binary variables. SMD is the standardized mean difference or Cohen's *d*, a measure of effect size. Balance was obtained (absolute value of SMD <= 0.1) on nearly all measures included in the propensity score model or the disease duration.

	Before Matching			After Matching		
Variable	TZ/DZ/AZ (*n* = 875)	Tamsulosin (*n* = 4,756)	SMD	TZ/DZ/AZ	Tamsulosin	SMD
**Demographics and Timing**						
Age (years)	72<br>(63, 80)	74<br>(64, 81)	-0.08	72<br>(63, 80)	72<br>(62, 81)	0.01
RX Start Year	2003<br>(2001, 2006)	2004<br>(2001, 2008)	-0.02	2004<br>(2001, 2006)	2004<br>(2002, 2008)	0.01
Lookback (years)	3.8<br>(2.2, 6.4)	4.8<br>(2.7, 7.8)	-0.26	4.3<br>(2.5, 6.8)	3.9<br>(2.3, 6.0)	0.19
Follow-Up (years)	1.8<br>(0.9, 4.1)	2.0<br>(0.9, 2.8)	-0.01	2.0<br>(0.9, 4.2)	1.9<br>(0.9, 4.1)	0.00
PD Disease Duration (years)	2.35<br>(1.53, 3.99)	2.74<br>(1.72, 4.60)	-0.18	2.57<br>(1.68, 4.25)	2.47<br>(1.66, 3.97)	0.10
**Baseline Healthcare Utilization**						
Inpatient Visits Per Year	0.14<br>(0.00, 1.34)	0.35<br>(0.00, 1.67)	-0.10	0.25<br>(0.00, 1.41)	0.13<br>(0.00, 1.48)	-0.13
Outpatient Visits Per Year	17<br>(10, 27)	17<br>(10, 27)	-0.01	17<br>(11, 28)	16<br>(9, 27)	0.01
Mean Number of Unique DX	0.35<br>(0.20, 0.62)	0.31<br>(0.17, 0.57)	0.05	0.31<br>(0.19, 0.56)	0.36<br>(0.21, 0.60)	-0.08
Outpatient DX Code Incidence	22<br>(14, 38)	25<br>(15, 42)	-0.10	23<br>(14, 39)	22<br>(12, 38)	0.00
**Elixhauser Comorbidities**						
Alcohol Abuse	12<br>(1.4%)	117<br>(2.5%)	-0.07	12<br>(1.6%)	13<br>(1.7%)	-0.01
Anemia	219<br>(25.0%)	1,204<br>(25.3%)	-0.01	201<br>(26.7%)	177<br>(23.5%)	0.07
Blood Loss	18<br>(2.1%)	130<br>(2.7%)	-0.04	16<br>(2.1%)	15<br>(2.0%)	0.01
Heart Failure	156<br>(17.8%)	923<br>(19.4%)	-0.04	138<br>(18.3%)	144<br>(19.1%)	-0.02
Coagulopathy	50<br>(5.7%)	309<br>(6.5%)	-0.03	46<br>(6.1%)	32<br>(4.2%)	0.09
Depression	143<br>(15.3%)	841<br>(17.7%)	-0.06	127<br>(16.8%)	106<br>(14.1%)	0.08
Diabetes W/O Complications	312<br>(35.7%)	1,545<br>(32.5%)	0.07	287<br>(38.1%)	247<br>(32.8%)	0.11
Diabetes W/ Complications	146<br>(16.7%)	752<br>(15.8%)	0.02	134<br>(17.8%)	103<br>(13.7%)	0.11
Drug Abuse	8<br>(0.9%)	81<br>(1.7%)	-0.06	6<br>(0.8%)	19<br>(2.5%)	-0.14
Fluid/Electrolyte Disorders	160<br>(18.3%)	993<br>(20.9%0	-0.06	147<br>(19.5%)	138<br>(18.3%)	0.03
HIV	3<br>(0.3%)	7<br>(0.1%)	0.05	3<br>(0.4%)	1<br>(0.1%)	0.05
HTN W/O Complications	623	3,310	0.04	555	528	0.08

	(71.2%)	(69.6%)		(73.6%)	(70.0%)	
HTN W/ Complications	169 (19.3%)	875 (18.4%)	0.02	159 (21.1%)	134 (17.8%)	0.08
Hypothyroidism	97 (11.1%)	641 (13.5%)	-0.07	95 (12.6%)	97 (12.9%)	-0.01
Liver Disease	38 (4.3%)	210 (4.4%)	0.00	36 (4.8%)	29 (3.8%)	0.05
Lymphoma	20 (2.3%)	94 (2.0%)	0.02	20 (2.7%)	13 (1.7%)	0.06
Metastatic Cancer	14 (1.6%)	120 (2.5%)	-0.06	14 (1.9%)	18 (2.4%)	-0.04
Other Neurological Disorders	626 (71.5%)	3,788 (79.6%)	-0.20	548 (72.7%)	511 (67.8%)	0.11
Obesity	45 (5.1%)	381 (8.0%)	-0.11	44 (5.8%)	33 (4.4%)	0.07
Paralysis	39 (4.5%)	267 (5.6%)	-0.05	36 (4.8%)	29 (3.8%)	0.05
Pulmonary/Circ Disorders	38 (4.3%)	244 (5.1%)	-0.04	35 (4.6%)	42 (5.6%)	-0.04
Psychoses	94 (10.7%)	568 (11.9%0	-0.04	89 (11.8%)	81 (10.7%)	0.03
Peptic Ulcer Disease	2 (0.2%)	22 (0.5%)	-0.04	2 (0.3%)	3 (0.4%)	-0.02
COPD	256 (29.3%)	1,536 (32.3%)	-0.07	226 (30.0%)	220 (29.2%)	0.02
Peripheral Vascular Disease	205 (23.4%)	1,329 (27.9%)	-0.10	193 (25.6%)	179 (23.7%)	0.04
Renal Failure	124 (14.2%)	507 (10.7%)	0.11	117 (15.5%)	108 (14.3%)	0.03
Rheumatoid Arthritis	44 (5.0%)	410 (8.6%)	-0.13	40 (5.3%)	40 (5.3%)	0.00
Solid Tumor	152 (17.4%)	991 (20.8%)	-0.09	130 (17.2%)	133 (17.6%)	-0.01
Valvular Disease	176 (20.1%)	1,193 (25.1%)	-0.12	170 (22.5%)	158 (21.0%)	0.04
Weight Loss	67 (7.7%)	501 (10.5%)	-0.10	57 (7.6%)	64 (8.5%)	-0.03
Prescribing Specific Considerations						
Indications						
PSA Measurement Taken	411 (47.0%)	1,998 (42.0%)	0.10	348 (46.2%)	343 (45.5%)	0.01
Abnormal PSA	109 (12.5%)	721 (15.2%)	-0.08	95 (12.6%)	103 (13.7%)	-0.03
Slow Urinary Stream	25 (2.9%)	139 (2.9%)	0.00	23 (3.1%)	19 (2.5%)	0.03
Uroflow Study	89 (10.2%)	424 (8.9%)	0.04	84 (11.1%)	62 (8.2%)	0.10
Cystometrogram	29 (3.3%)	118 (2.5%)	0.05	25 (3.3%)	21 (2.8%)	0.03
Diagnosis of BPH	365 (41.7%)	2,043 (43.0%)	-0.03	323 (42.8%)	305 (40.5%)	0.05
Adverse Event Risks						
Orthostatic Hypotension	28 (3.2%)	276 (5.8%)	-0.12	27 (3.6%)	35 (4.6%)	-0.05
Other Hypotension	45 (5.1%)	375 (7.9%)	-0.10	41 (5.4%)	35 (4.6%)	-0.04

7
